# Mass Defect Filtering-Oriented Identification of Resin Glycosides from Root of *Convolvulus scammonia* Based on Quadrupole-Orbitrap Mass Spectrometer

**DOI:** 10.3390/molecules27113638

**Published:** 2022-06-06

**Authors:** Qiang Yin, Rahima Abdulla, Gulmira Kahar, Haji Akber Aisa, Chunting Li, Xuelei Xin

**Affiliations:** 1 State Key Laboratory Basis of Xinjiang Indigenous Medicinal Plants Resource Utilization, Xinjiang Technical Institute of Physics and Chemistry, Chinese Academy of Sciences, Urumqi 830011, China; yinqiang@renfu.com.cn (Q.Y.); rahima@ms.xjb.ac.cn (R.A.); 13999291054@163.com (G.K.); haji@ms.xjb.ac.cn (H.A.A.); 2 University of Chinese Academy of Sciences, Beijing 100049, China

**Keywords:** mass defect filter, resin glycosides, glycosidic acids, *Convolvulus scammonia*

## Abstract

This work aimed to develop and evaluate a post-acquisition data processing strategy, referred to as a mass defect filter (MDF), for rapid target the resin glycosides in root of *Convolvulus scammonia* by setting mass rang and mass defect range from high-resolution MS data. The full-scan mass data were acquired by high-performance liquid chromatography coupled with Q Exactive Plus hybrid quadrupole-orbitrap mass spectrometer that featured high resolution, mass accuracy, and sensitivity. To screen resin glycosides, three parent filter *m*/*z* 871, *m*/*z* 853, and *m*/*z* 869 combined with diagnostic fragment ions (DFIs) approach were applied to remove the interference from complex herbal extract. The targeted components were characterized based on detailed fragment ions. Using this approach, 80 targeted components, including 22 glycosidic acids and 58 resin glycosides were tentatively identified. The present results suggested that the proposed MDF strategy would be adaptable to the analysis of complex system in relevant filed.

## 1. Introduction

*Convolvulus scammonia* belongs to morning glory family (Convolvulaceae) and its extractive scammony, known commonly as resin glycosides which are unique secondary metabolites in natural. These active principles are responsible for the drastic purgative action and Convolvulaceous plants used in traditional medicine throughout the world, especially *Convolvulus scammonia* [1].

A new interest in resin glycosides caused by the discovery of novel biological activities such as cytotoxicity toward cancer cells [1,2], anti-bacterial [1], anti-viral [3,4,5], anti-inflammatory [6], and multidrug resistance modulatory [7,8].

Resin glycosides are composed of differently acylated oligosaccharides glycosidically linked to hydroxylated fatty acids which are usually linked back to the sugar chain to form macro lactone rings of various sizes [1,9]. Even though phytochemical investigations on *Convolvulus scammonia* were initiated in the last nineteenth century, scammonic acid A together with scammonins I–VIII have been discovered [10,11,12], the structure of more active compounds still remain unrevealed. Due to the pivotal role in preventing and curing disease, the structural features of resin glycosides in *Convolvulus scammonia* should be investigated further. However, the structural complexity, high molecular weight, and lack of references render characterization and identification of resin glycosides challenging.

A hybrid quadrupole-orbitrap mass spectrometer (Q-Orbitrap) is currently a preferred platform for chemical elucidation [13,14,15,16,17], combined with data mining strategies, such as mass defect filter (MDF) [18,19], neutral loss filter (NLF), and diagnostic ion filter (DIF) which have exhibited superiority in characterization of the interested components.

To sensitively characterize the resin glycosides in *Convolvulus scammonia*, we present a strategy that enable targeted components characterization established on a mass defect filter (MDF). The core of this strategy is setting the mass tolerance and mass defect tolerance based on high-resolution mass spectrometry data and then excluding ions that fall out of the expected range to obtain the mass spectrum data set for the target compounds [20,21].

By this strategy, 80 targeted components were tentatively characterized, including 22 glycosidic acids and 58 resin glycosides, among which minor components are screened and characterized for the first time in *Convolvulus scammonia*. The unprecedented one *m*/*z* 1149 is another case of resin glycoside with a hydroxy C_17_ fatty acid as its aglycone [22].

## 2. Results and Discussion

### 2.1. DFIs Determinations and Fragmentation Patterns Analysis

DFIs was originally proposed from the fact that the components contained in TCMs could usually be structurally classified into several subfamilies and components of the same family usually contained the same carbon skeleton or substructures, from which the same fragment ions could be defined as diagnostic fragment ions (DFIs). Then, these well-defined DFIs were used to screen and identify the analogues. Herein we applied such a strategy to analysis with predefined diagnostic fragment ions [23].

Turpethic acids C and Turpethoside B were used as the references compounds to deduce the fragmentation of resin glycosides and glycosidic acids. In the MS/MS spectrum, the observed neutral loss (NL) masses and diagnostic fragment ions (DFIs) in support of the characterization of glycosidic acids and resin glycosides are offered in Table 1.

**Figure 1:**
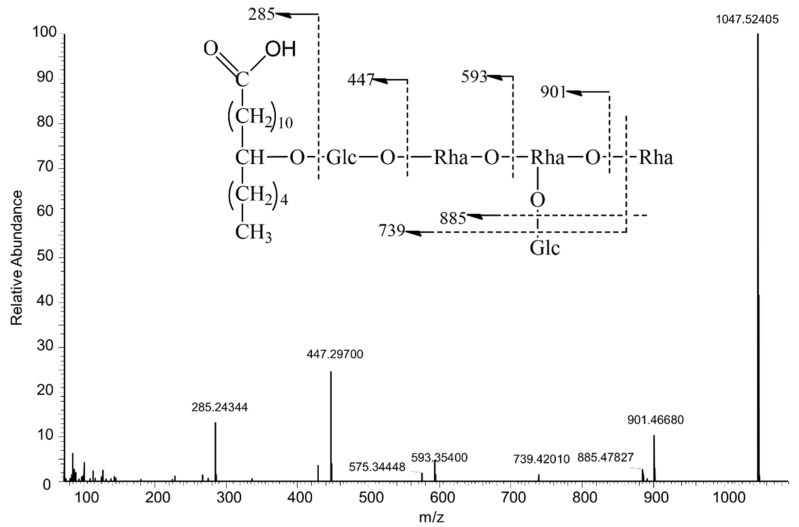
Proposed fragmentation pathway of Turpethic acids C.

Turpethic acids C (Figure 1), a glycosidic acid with deprotonated ion [M − H]^−^ at *m*/*z* 1047, neutral loss of rhamnopyranosyl (146 Da, Rha) generate fragment ion *m*/*z* 901, loss of glucopyranosyl (162 Da, Glc) made ion *m*/*z* 885, *m*/*z* 739 [M − H − 146 − 162]^−^, *m*/*z* 593 [739 − C_6_H_10_O_4_]^−^ combined with *m*/*z* 447 [593 − C_6_H_10_O_4_]^−^ were checked. Characteristic ion *m*/*z* 285 [447 − C_6_H_10_O_5_]^−^, which was 14 Da higher than jalapinolic acid [(S)-11-hydroxyhexadecanoic acid] indicated this resin glycoside having a hydroxylated C_17_ fatty acid as its aglycone.

**Figure 2:**
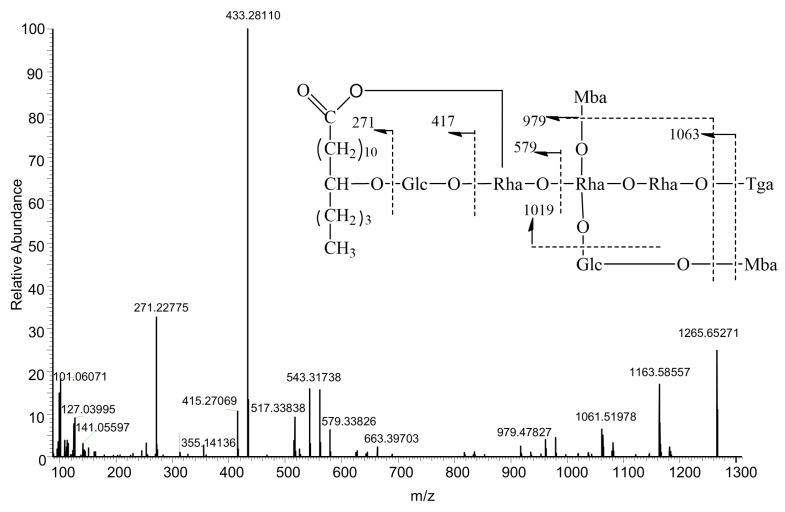
Proposed fragmentation pathway of Turpethoside B.

Turpethoside B exhibited [M − H]^−^ at *m*/*z* 1265 (Figure 2), fragmentation ion at *m*/*z* 1163 was attributed to loss of 2-methylbutyric acid (102 Da, Mba) from *m*/*z* 1265 [M − H − C_5_H_10_O_2_]^−^, successively loss of 2-methylbutyric acid and tiglic acid (100 Da, Tga) to produce *m*/*z* 1063 [1265 − C_5_H_8_O_2_ − C_5_H_10_O_2_]^−^, *m*/*z* 1061 [1265 − C_5_H_10_O_2_ − C_5_H_10_O_2_]^−^, *m*/*z* 1019 [1265 − C_11_H_18_O_6_ (O-methylbutyrylglucose unit)]^−^ *m*/*z* 979 [1265 + H_2_O − C_5_H_10_O_2_ − C_5_H_10_O_2_ − C_5_H_8_O_2_]^−^, *m*/*z* 961 [1265 − C_5_H_10_O_2_ − C_5_H_10_O_2_ − C_5_H_8_O_2_]^−^ together with diagnostic fragment ion *m*/*z* 835 [1265 + H_2_O − C_11_H_18_O_6_ − C_5_H_8_O_2_ − C_5_H_10_O_2_]^−^, *m*/*z* 579 [835 − 146 − (146 − 2 × H_2_O)]^−^, *m*/*z* 561 [835 − 146 − (146 − H_2_O)]^−^, *m*/*z* 543 [835 − 146 − 146]^−^, *m*/*z* 433 [579 − 146]^−^, *m*/*z* 417 [579 − 162]^−^ as well as *m*/*z* 271 [417 − 146]^−^ were observed.

### 2.2. Construction of MDF Model

**Figure 3:**
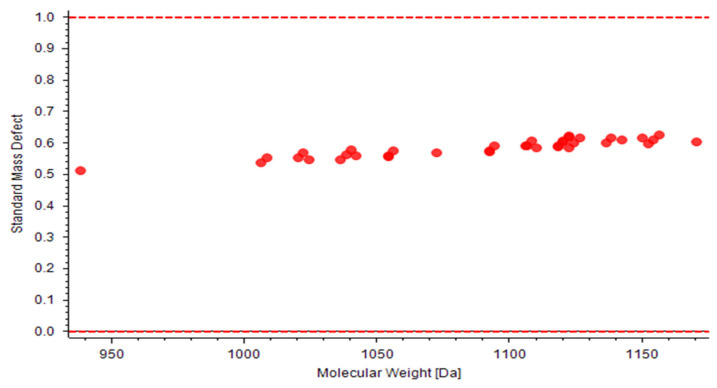
Targeted compounds screened from *Convolvulus scammonia* by MDF.

According to resin glycosides in Convolvulus scammonia were reported. Jalapinolic acid [(S)-11-hydroxyhexadecanoic acid] is the most frequently aglycone for the macrocyclic lipooligosaccharide core. Therefore, the parent drug filter was based on the jalapinolic acid, *m*/*z* 871 (C_40_H_71_O_20_) picked as filter for glycosidic acids, *m*/*z* 853 (C_40_H_69_O_19_) and *m*/*z* 869(C_40_H_69_O_20_) for resin glycosides. Transformations such as 2-methylbutyric acid (Mba), tiglic acid (Tga), isobutyric acid (Iba), (2R,3R)-3-hydroxy-2-methylbutyric (Nia), 3-hydroxy-methylenebutyric (Hma) were supplied to MDF workflow. Using the mass defect values calculated above and the exact mass values calculated from the elemental composition input (Table 2), mass tolerance 112 Da and mass defect tolerance 0.058 Da were set to define the rectangular mass defect filters (Table 3). The use of the MDF to filtrate targeted compounds from Convolvulus scammonia are given in Figure 3.

**Figure 4:**
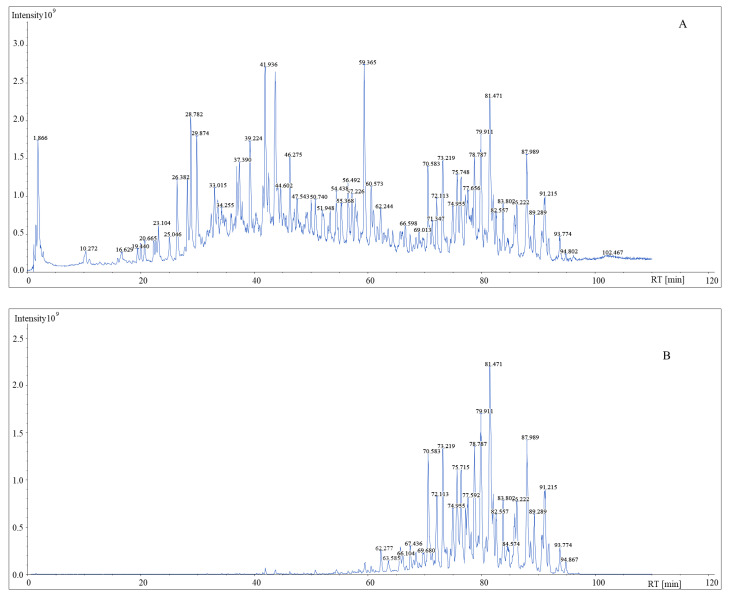
The TIC chromatogram of *Convolvulus scammonia* without filtration (**A**) and with MDF filtration (**B**).

The generated chromatograms are displayed in Figure 4. As shown in the chromatogram, after filtration by MDF technique, the noise level was lower than the original chromatogram and ions which the mass defect were not within the filter ranges, were excluded, the targeted components became predominant ions in the TIC. Meanwhile, the sensitivity of minor constituents such as *m*/*z* 1170.60286 at 59.46 min was largely improved.

### 2.3. Identified Glycosidic Acids

Glycosidic acids exhibited deprotonated ions as the base peak were observed; neutral loss of monosaccharide units including deoxyhexose, hexose and short organic acid moieties such as tiglic acid, 2-methylbutyric acid, (2R,3R)-3-hydroxy-2-methylbutyric were the main fragment patterns; aglycone ions *m*/*z* 257, *m*/*z* 271, *m*/*z* 285 indicated the presence of the hydroxylated fatty acid with chain lengths of C_15_, C_16_, and C_17_, respectively. A total of 22 glycosidic acids were detected and tentatively characterized in root of *Convolvulus scammonia*, in which 21 compounds were based on scammonic acid A parent filter, Their ESI-MS^n^ information is shown in Table 4. Notable ion at *m*/*z* 1170 was founded that scammonic acid B as its parent drug. Diagnostic fragment ion at *m*/*z* 271 was the common aglycone, suggesting the presence of jalapinolic acid.

Peak 1, 5, 8 all gave high intensity of deprotonated ion at *m*/*z* 1053,the HCD-MS^2^ of which exhibited produced ions at *m*/*z* 953 [M − H − 100(3hydroxy-2-methylbutyric acid residue)]^−^, *m*/*z* 871 [M − H − 100(3-hydroxy-2-methylbutyric acid residue) − 82(tiglic acid residue)]^−^, which indicated the parent drug filter was scammonia acid A, diagnostic fragment ions *m*/*z* 853 [1053 + H_2_O − 118 − 100]^−^, *m*/*z* 835 were generated due to successively loss of 3-hydroxy-2-methylbutyric acid and tiglic acid from *m*/*z* 1053, furthermore, successive elimination of deoxyhexose moiety (146 Da), hexose moiety (162 Da) to generate *m*/*z* 725 [871 − 146]^−^, *m*/*z* 579 [725 − 146 − 146]^−^, *m*/*z* 417 [579 − 162]^−^ coupled with *m*/*z* 271 [M − H − 100 − 82 − 146 − 146 − 162 − 146]^−^ supported the existence of scammonic acid A. It was conclusion that peak 1, 5, 8 were isomer of 3-hydroxy-2-methylbutyric acid + tiglic acid + scammonic acid A.

Peak 2, 6 had the same [M − H]^−^ ion at *m*/*z* 1055, which was tentatively identified as isomer of 3-hydroxy-2-methylbutyric acid + 2-methylbutyric acid + scammonic acid A. The typical fragment ions *m*/*z* 955 [M − H − 100]^−^, *m*/*z* 871 [M − H − 100 − 84(2-methylbutyric acid residue)]^−^, *m*/*z* 853 [M − H + H_2_O − 118 − 102]^−^ represented successively loss of 3-hydroxy-2-methylbutyric acid residue (100 Da), 2-methylbutyric acid residue (84 Da) from deprotonated ion, respectively. It’s also gave ions at *m*/*z* 579 (loss of 2 mol deoxyhexose moiety, 146 Da), *m*/*z* 417 (loss of hexose moiety, 162 Da) and *m*/*z* 271 in MS/MS spectrum by cleavage of glycosidic bond on *m*/*z* 871(scammonic acid A).

Peak 3 was eluted at tR 59.405 min with precursor ion at *m*/*z* 1169, its diagnostic ions *m*/*z* 887 of scammonic acid B can distinguished itself from scammonic acid A (*m*/*z* 871). Fragment ions at *m*/*z* 969 [1169 − 100(3-hydroxy-2-methylbutyric acid residue) − 100]^−^, *m*/*z* 951 [1169 − 100 − 82(tiglic acid residue)]^−^, *m*/*z* 887 [969 − 82]^−^ were attributed to loss of C_5_H_6_O residue (tiglic acid residue, 82 Da) from *m*/*z* 969, *m*/*z* 851 [951 − 100]^−^ were also detected. Characteristic ions *m*/*z* 579 were observed for elimination of deoxyhexose moiety 146 Da and hexose moiety 162 Da from scammonic acid B (*m*/*z* 887), in addition, *m*/*z* 417 [579 − 162]^−^ combined with *m*/*z* 271 [417 − 146]^−^ supported that oligoglycosides conjunction type in scammonic acid B was deoxyhexose–hexose–deoxyhexose–hexose. According to information mentioned above, peak 3 was tentatively characterized as 3-hydroxy-2-methylbutyric acid + 3-hydroxy-2-methylbutyric acid + tiglic acid + scammonic acid B.

Peak 4 had high intensity ion at *m*/*z* 1071 with elemental composition C_50_H_87_O_24_, *m*/*z* 971 indicated the transformation was 3-hydroxy-2-methylbutyric acid residue [M − H − 100]^−^, scammonic acid A as parent drug template was supported from diagnostic ions *m*/*z* 871 [971 − 100]^−^, *m*/*z* 853 [1071 + H_2_O −118 − 118]^−^, *m*/*z* 725 [871 − 146(deoxyhexose moiety)]^−^, *m*/*z* 579, *m*/*z* 561 and *m*/*z* 417. It supposed that peak 4 was 2 mol 3-hydroxy-2-methylbutyric acid substituted on scammonic acid A.

Peak 7 gave deprotonated ion at *m*/*z* 1041 eluted at retention time of 65.234, fragmentation ions at *m*/*z* 941 which was attributed to loss of 3-hydroxy-2-methylbutyric acid residue (100 Da) from *m*/*z* 1041, *m*/*z* 923 [1041 − 118]^−^. Typical ions at *m*/*z* 871 [M − H − 100(3-hydroxy-2-methylbutyric acid resiue) − 70(isobutyric acid residue)]^−^, *m*/*z* 853 [1041 + H_2_O − 118 − 88]^−^, *m*/*z* 835 [1041 − 118 − 88]^−^ as evidence for the presence of scammonic acid A. In addition, *m*/*z* 579, *m*/*z* 417 as well as *m*/*z* 271 supported that. The tentative identification given to peak 7 was 3-hydroxy-2-methylbutyric acid + isobutyric acid + scammonic acid A.

Peak 9, 10, 11, 12, 13 were tentatively characterized as isomer of 3-hydroxy-2-methylbutyric acid + 3-hydroxy-2-methylbutyric acid + tiglic acid + scammonic acid A according to elemental composition C_55_H_94_O_25_ (MW, 1154 Da). Fragmentation ions at *m*/*z* 1053 [M − H − 100]^−^, *m*/*z* 953 [M − H − 100 − 100]^−^ demonstrated that the existence of 3-hydroxy-2-methylbutyric acid, successive loss of tiglic acid to give fragmentation ion at *m*/*z* 871 which was diagnostic ion of scammonic acid A. furthermore, *m*/*z* 579, *m*/*z* 417, *m*/*z* 217 were founded in MS/MS spectrum.

Peak 14, 16 as well as 19 displayed the same deprotonated ion at *m*/*z* 1123, which was 100 Da higher than *m*/*z* 1023, it indicated that the presence of 3-hydroxy-2-methylbutyric acid. In addition to this, there were similarity fragmentation ions with *m*/*z* 1023 [M − H − 100(3-hydroxy-2-methylbutyric acid residue)]^−^, such as *m*/*z* 941, *m*/*z* 871, *m*/*z* 579, *m*/*z* 417, *m*/*z* 271. Above all, we inferred that ion at *m*/*z* 1123 was based on parent filter scammonic acid A and substituted by 3-hydroxy-2-methylbutyric acid, tiglic acid as well as isobutyric acid.

Peak 15 showed [M − H]^−^ ions at *m*/*z* 1023 with the elemental composition of C_49_H_84_O_22_. In the MS/MS spectrum, *m*/*z* 941 indicated that loss of substitutes tiglic acid residue (82 Da) from [M − H]^−^, and then successively loss of isobutyric acid residue (70 Da) to produce ions at *m*/*z* 871, fragmentation ions *m*/*z* 725, *m*/*z* 579 suggested successive loss of rhamnose monohydrate from *m*/*z* 871, neutral loss of glucose (162 Da) to produce ion at *m*/*z* 417. Diagnostic ion *m*/*z* 271 supplied the evidence for the existence of jalapinolic acid aglycone. We inferred from fragmentation ions that peak 15 was scammonic acid A combined with 1 mol each of tiglic acid and isobutyric acid.

Peak 17 and 22 exhibited [M − H]^−^ ion at *m*/*z* 1137. Prominent ion at *m*/*z* 871 [1137 − 82(tiglic acid residue) − 100(3-hydroxy-2-methylbutyric acid residue − 84(2-methylbutyric acid residue)]^−^ provided evidence for the existence of scammonic acid A. Fragmentation ions at *m*/*z* 1055, *m*/*z* 955, *m*/*z* 937 [1137 + H_2_O − 100 − 118]^−^, *m*/*z* 835 [1137 + H_2_O − 100 − 118 − 102]^−^ were checked. Breakage of glycosidic bond was the main fragmentation pathway of scammonic acid A, from which *m*/*z* 579 [871 − 146 − 146]^−^, *m*/*z* 417 [871 − 146 − 146 − 162]^−^ and *m*/*z* 217 [871 − 146 − 146 − 162 − 146]^−^ were generated. We presumed that peak 17 and 22 were isomer of scammonic acid A + tiglic acid + 3-hydroxy-2-methylbutyric acid + 2-methylbutyric acid.

Peak 18, the [M − H]^−^ *m*/*z* 1037 with formula C_50_H_86_O_22_ eluted at 74.561 min, in analysis of its MS/MS spectrum, the [M − H − 82(tiglic acid residue)]^−^ ion at *m*/*z* 955, *m*/*z* 935 [M − H − 102(2-methylbutyric acid)]^−^, [M − H − 82 − 84(2-methylbutyric acid resiude)]^−^ ion at *m*/*z* 871 and characteristic ions of scammonic acid A were checked. Compared with peak 18, 23, peak 19 was 100 Da lower, it illustrated that there was absence of 3-hydroxy-2-methylbutyric acid. Therefore, we inferred that peak 18 was based on scammonic acid A and substituted by tiglic acid and 2-methylbutyric acid.

Peak 20 showed [M − H]^−^ at *m*/*z* 1039, fragmentation ions at *m*/*z* 955, *m*/*z* 871 demonstrated that 2 mol of 2-methylbutyric acid substituted on scammonic acid A, *m*/*z* 579, *m*/*z* 417 combined with *m*/*z* 271 as notable markers for scammonic acid A were checked in MS/MS spectrum. Peak 20 was tentatively characterized as scammonic acid A + 2-methylbutyric acid + 2-methylbutyric acid.

Peak 21 with high intensity at *m*/*z* 1125 was eluted at 78.93min. Study of the MS/MS spectrum of glycosidic acids, loss of organic acid and cleavage of glycosidic bond were main fragmentation pathway, ions at *m*/*z* 1025 [M − H − 100]^−^, *m*/*z* 941 [M − H − 100 − 84]^−^ as well as *m*/*z* 871 [M − H − 100 − 84 − 70]^−^ were generated according to successively loss of 3-hydroxy-2-methylbutyric acid, 2-methylbutyric acid and isobutyric acid from deprotonated ion *m*/*z* 1125. Diagnostic ions of scammonia acid A at *m*/*z* 579, *m*/*z* 417, *m*/*z* 271 were observed. We presumed that peak 21 was scammonic acid A + 3-hydroxy-2-methylbutyric acid + 2-methylbutyric acid and isobutyric acid.

### 2.4. Identified Resin Glycosides

Resin glycosides in *Convolvulus* *scammonia* L share the common structure of a macrolactone composed by one acylated glycosidic acid. Through the post-data mining strategy MDF, totally 58 resin glycosides were checked, in which 56 resin glycosides were established on parent filter *m*/*z* 853 except ion at *m*/*z* 1151 and *m*/*z* 1149.

Peak was eluted at 85.271min with elemental composition C_56_H_94_O_20_. The precursor ion [M − H]^−^ *m*/*z* 1149 firstly elimination of C_2_H_4_O (44 Da), fragmentation ion *m*/*z* 1049 was attributed to loss of 100(3-hydroxy-2-methylbutyric acid residue) from precursor ion. Ion at *m*/*z* 949 [1049 − 100]^−^ indicated there was existence of 2 mol 3-hydroxy-2-methylbutyric acid in the structure. Diagnostic fragment ion *m*/*z* 867 [1049 − 100 − 82(tiglic acid residue)]^−^ coupled with noteworthy ions *m*/*z* 849, *m*/*z* 593 [867 − 146 − (146 − H_2_O)]^−^, *m*/*z* 575 [867 − 146 − 146]^−^, *m*/*z* 431 [593 − 162]^−^, *m*/*z* 413 [575 − 162]^−^ as well as *m*/*z* 285 [431 − 146]^−^ were checked. These ions showed the evidence for the existence of C_17_ fatty acid aglycone. According to the detailed fragmentation ion information and fragment rules, we inferred that compound with structural of *m*/*z* 867 + 2 mol 3-hydroxy-2-methylbutyric acid + tiglic acid.

Peak with elemental composition C_55_H_92_O_25_ (*m*/*z* 1151) was eluted at 71.689 min. In MS/MS spectrum [M + HCOO]^−^ ion at *m*/*z* 1197 was observed, its deprotonated ion at *m*/*z* 1151 successively loss of 3-hydroxy-2-methylbutyric acid (118 Da) to generate ion at *m*/*z* 1051 [M − H − 100(3-hydroxy-2-methylbutyric acid residue)]^−^ and *m*/*z* 951 [M − H − 100]^−,^ respectively. Fragmentation ion *m*/*z* 1033 [1151 − 118]^−^, 1007 [1151 − 144(hexose unit − H_2_O)]^−^, *m*/*z* 933 [1151 + H_2_O − 118 − 118]^−^ coupled with *m*/*z* 907 [1151 − C_11_H_16_O_6_(O-tigloylhexose unit)]^−^ and *m*/*z* 905 [1151 − C_11_H_18_O_6_(O-3-hydroxy-2-methylbutyrylpentose unit)]^−^ were checked. Diagnostic ion at *m*/*z* 869 [M − H − 100 − 100 − 82(tiglic acid residue)]^−^ demonstrated the sugar units were hexose–pentose–pentose–hexose which linked to jalapinolic acid to form macrolactone ring, it also illustrated the existence of tiglic acid. According to the fragmentation ions, we inferred this compound was based on parent drug filter *m*/*z* 869 and substituted by 2 mol 3-hydroxy-2-methylbutyric acid and 1 mol tiglic acid.

Peaks listed below were all based on parent template *m*/*z* 853, totally 56 peaks were tentatively characterized, the detail of ESI-MS^n^ information is shown in Table 5. Analysis of the MS/MS spectrum, the proposed fragmentation patterns of resin glycosides are as follows:

- adduct ion [M + HCOO]^−^, [M + Cl]^−^ combined with [M − H]^−^ were observed, it is different from glycosidic acids;
- loss of short organic acid such as 2-methylbutyric acid, tiglic acid, isobutyric acid, (2R,3R)-3-hydroxy-2-methylbutyric, 3-hydroxy-2-methylenebutyric were common characteristics;
- breakage of glycosidic linkage is prone to loss of 162 Da, 146 Da;
- loss of C_2_H_4_O (44 Da) was observed when (2R,3R)-3-hydroxy-2-methylbutyric substituted on resin glycosides
- parent drug filter ion at *m*/*z* 853 with lower intensity was obtained and identical ions at *m*/*z* 579, *m*/*z* 561, *m*/*z* 543, *m*/*z* 399, *m*/*z* 417 combined with *m*/*z* 271 were discovered.

Peak 1′ displayed precursor ion at *m*/*z* 937 and adduct ion [M + HCOO]^−^ at *m*/*z* 983, [M + Cl]^−^ at *m*/*z* 973 were found. Diagnostic ion at *m*/*z* 853 [M − H − 84(2-methylbutyric acid residue)]^−^, *m*/*z* 835 [937 − 102]^−^ were discovered in MS/MS spectrum, from which we inferred that there was 2-methylbutyric acid substituted on parent drug filter *m*/*z* 853. Therefore, Peak 1′ was tentatively identified as *m*/*z* 853 + 2-methylbutyric acid.

Peak 2′, 5′, 6′, 8′, 11′, 13′, 22′ all exhibited [M + HCOO]^−^ at *m*/*z* 1081 with high intensity and [M + Cl]^−^ at *m*/*z* 1071 in MS spectrum, analysis of MS/MS spectrum, [M − H]^−^ at *m*/*z* 1035 (C_50_H_83_O_22_) gave high abundance, fragmentation ion at *m*/*z* 991 [M − H − 44]^−^, *m*/*z* 953 [1035 − 82(tiglic acid residue)]^−^ and *m*/*z* 935 [1035 − 100(3-hydroxy-2-methylbutyric)]^−^ with highest intensity appeared. Diagnostic ion *m*/*z* 853 [935 − 82(tiglic aid residue)]^−^ with ion *m*/*z* 835 [1035 + H_2_O − 118 − 100]^−^ served as evidence for parent drug. Based on fragmentation rules, peak 2′, 5′, 6′, 8′, 11′, 13′, 22′ were characterized as isomer of *m*/*z* 853 + 3-hydroxy-2-methylbutyric + tiglic aid.

Peak 3′ showed [M + HCOO]^−^ at *m*/*z* 1069 and [M − H]^−^ at *m*/*z* 1023 in MS/MS spectrum. Ion at *m*/*z* 979 indicated loss of 44 Da (C_2_H_4_O) from deprotonated ion, *m*/*z* 935 through loss of isobutyric acid from *m*/*z* 1023, *m*/*z* 923 [M − H − 100]^−^ demonstrated the presence of (2R,3R)-3-hydroxy-2-methylbutyric, noteworthy ion at *m*/*z* 835 [M − H − 100 − 88]^−^ as parent drug filter observed. A tentative identification of Peak 3′ was *m*/*z* 853 + (2R,3R)-3-hydroxy-2-methylbutyric + isobutyric acid.

Peak 4′, 20′, 25′ all gave [M − H]^−^ at *m*/*z* 1123 was 100 Da higher than *m*/*z* 1023, which had the same fragmentation pattern with *m*/*z* 1023, namely loss of organic acid and breakage of glycosidic linkage. From fragmentation ions *m*/*z* 1079, *m*/*z* 1035, *m*/*z* 1023 as well as *m*/*z* 835, we presumed the structure was *m*/*z* 853 + 2 mol 3-hydroxy-2-methylbutyric + isobutyric acid.

Peak 7′, 10′, 16′, 18′ all exhibited [M + HCOO]^−^ at *m*/*z* 1083 and [M + Cl]^−^ at *m*/*z* 1073 in MS spectrum, analysis of MS/MS spectrum [M − H]^−^ at *m*/*z* 1037 combined with *m*/*z* 993 [M − H − 44]^−^, *m*/*z* 937 [M − H − 100(3-hydroxy-2-methylbutyric residue)]^−^ as well as markable fragmentation ion at *m*/*z* 853 [937 − 84]^−^, *m*/*z* 579, *m*/*z* 561, *m*/*z* 417, *m*/*z* 217 were checked. Peak 7′, 10′, 16′, 18′ were tentatively identified as isomer of *m*/*z* 853 + 3-hydroxy-2-methylbutyric + 2-methylbutyric acid.

Peak 9′, 14′ showed [M + HCOO]^−^ at *m*/*z* 1099 with high abundance. In analysis of MS/MS spectrum, [M − H]^−^ at *m*/*z* 1053 with elemental composition C_50_H_85_O_23_ was prone to loss of 44 Da to form ion at *m*/*z* 1009, ions at *m*/*z* 909 and *m*/*z* 891 were formed from [M − H − 44 − 100(3-hydroxy-2-methylbutyric residue)]^−^, [M − H − 44 − 118]^−^, respectively. Fragmentation ions at *m*/*z* 953 [M − H − 100]^−^, *m*/*z* 853 [M − H − 100 − 100]^−^, *m*/*z* 835 [1053 + H_2_O − 118 − 118]^−^ both of these fragmentation ions illustrated the existence of 2 mol 3-hydroxy-2-methylbutyric. According to the fragment rules, peak 9′ and 14′ were tentatively characterized as isomer of *m*/*z* 853 + 2 mol 3-hydroxy-2-methylbutyric.

Peak 12′, 15′, 17′, 19′, 21′, 23′, 26′, 28′, 41′ were tentatively identified as isomer of *m*/*z* 853 + 2 mol 3-hydroxy-2-methylbutyric + tiglic acid according to fragmentation rule of resin glycosides. The [M + HCOO]^−^ ion at *m*/*z* 1181 with high intensity and [M + Cl]^−^ ion at *m*/*z* 1171 were observed in MS spectrum, in analysis of its MS/MS spectrum, the noteworthy ions *m*/*z* 1091 and *m*/*z* 1047 were due to successively loss of C_2_H_4_O (44 Da) from precursor ion *m*/*z* 1135, ion at *m*/*z* 1035 was attributed to loss of 100 Da (3-hydroxy-2-methylbutyric residue) from [M − H]^−^, *m*/*z* 991 [1091 − 100]^−^, *m*/*z* 935 from [M − H − 100 − 100]^−^. Characteristic ion *m*/*z* 853 [M − H − 100 − 100 − 82]^−^ was checked, it illustrated the existence of tiglic acid, diagnostic ions of at *m*/*z* 579, *m*/*z* 561, *m*/*z* 417 combined with *m*/*z* 217 supported the parent drug filter was *m*/*z* 853.

Peak 24′, 29′, 32′, 34′ showed [M − H]^−^ at *m*/*z* 1137, successively loss of 44 Da from *m*/*z* 1137 to generate *m*/*z* 1093 and *m*/*z* 1049 with high abundance, according to fragmentation pattern of *m*/*z* 1135 we inferred there were 2 mol 3-hydroxy-2-methylbutyric substituted on resin glycosides. Ion at *m*/*z* 1037 [1137 − 100(3-hydroxy-2-methylbutyric residue]^−^, *m*/*z* 991 [1093 − 2-methylbutyric acid], *m*/*z* 853 [1037 − 100 − 84]^−^, *m*/*z* 579, *m*/*z* 561, *m*/*z* 417 as well as *m*/*z* 271 were discovered. Therefore, peak 24′, 29′, 32′, 34′ were tentatively characterized as *m*/*z* 853 + 2 mol 3-hydroxy-2-methylbutyric + 2-methylbutyric acid.

Peak 27′, 30′, 36′, 42′, 46′, 54′ exhibited high intensity [M + HCOO]^−^ at ion *m*/*z* 1151 and [M + Cl]^−^ at ion *m*/*z* 1141 also checked in MS spectrum. [M − H]^−^ at ion *m*/*z* 1105 loss of 44 Da to make fragmentation ion at *m*/*z* 1061, ion at *m*/*z* 1005 from deprotonated ion *m*/*z* 1105 dropped 3-hydroxy-2-methylbutyric residue (100 Da), *m*/*z* 973 [1061 − 88(isobutyric acid)]^−^, *m*/*z* 961 [1061 − C_5_H_8_O_2_(tiglic acid)]^−^, *m*/*z* 917 [1005 − isobutyric acid]^−^, *m*/*z* 835 [917 − (tiglic acid − H_2_O)]^−^ were found. Cleavage of glycoside bond was prone to produce diagnostic ions *m*/*z* 579, *m*/*z* 561, *m*/*z* 417 and *m*/*z* 271. We presumed that the structure of peak 27′, 30′, 36′, 42′, 46′, 54′ was 3-hydroxy-2-methylbutyric, tiglic acid combined with isobutyric acid substituted on parent drug *m*/*z* 853.

Peak 31′, 48′, 51′ displayed [M + HCOO]^−^ at *m*/*z* 1153 and [M + Cl]^−^ at *m*/*z* 1143. In the MS/MS spectrum [M − H]^−^ at *m*/*z* 1107 with elemental composition C_54_H_91_O_23_ was observed, *m*/*z* 1005 was attributed to loss of 2-methylbutyric acid from 1107, *m*/*z* 989 [M − H − 118]^−^ and *m*/*z* 905 [M − H − 102 − 100]^−^ illustrated the presence of 3-hydroxy-2-methylbutyric. Cleavage of glycoside bond and loss of hexose moiety, pentose moiety as noteworthy fragment pathway was checked, the corresponding fragment ions at *m*/*z* 579 [835 − 146 − (146 − 2 × H_2_O)]^−^, *m*/*z* 561 [835 − 146 − (146 − H_2_O)]^−^, *m*/*z* 543 [835 − 146 − 146]^−^, *m*/*z* 399 [561 − 162]^−^, *m*/*z* 417 [579 − 162]^−^ combined with *m*/*z* 271 [417 − 146]^−^ were discovered. Peak 31′, 48′, 51′ were tentatively characterized as isomer of *m*/*z* 853 + 2-methylbutyric acid + 3-hydroxy-2-methylbutyric + isobutyric acid based on fragment rules.

Peak 33′, 38′ gave [M + HCOO]^−^ at *m*/*z* 1053 and [M − H]^−^ at *m*/*z* 1007 which was 100 Da lower than *m*/*z* 1107, it presumed absence of 3-hydroxy-2-methylbutyric. Fragment ion at *m*/*z* 923 [M − H − 84]^−^, *m*/*z* 905 [M − H − 102]^−^ suggested the existence of 2-methylbutyric acid, ion at *m*/*z* 919 [M − H − 88]^−^ coupled with *m*/*z* 835 [M − H − 102 − 70]^−^ demonstrated the presence of isobutyric acid. The diagnostic ions *m*/*z* 579, *m*/*z* 561, *m*/*z* 417, *m*/*z* 399 as well as *m*/*z* 271 supported the breakage of glycosides bond from *m*/*z* 853. Therefore, the tentative identification given to peak 33′, 38′ was isomer of *m*/*z* 853 + 2-methylbutyric acid+ isobutyric acid.

Peak 35′, 40′ were tentatively characterized as isomer of *m*/*z* 853 + 2-methylbutyric acid + tiglic acid based on proposed fragment rules of resin glycosides. A high abundance of [M + HCOO]^−^ ion at *m*/*z* 1065 coupled with [M + Cl]^−^ at *m*/*z* 1054 were discovered. In analysis of MS/MS spectrum, the precursor ion at *m*/*z* 1019 gave high intensity, ion at *m*/*z* 937 [M − H − 82]^−^, *m*/*z* 919 [M − H − 100]^−^ served as evidence for the existence of tiglic acid. Ion at *m*/*z* 853 [M − H − 82 − 84]^−^ demonstrated the presence of 2-methylbutyric acid.

Peak 37′, 49′, 52′, 56′ showed [M − H]^−^ at *m*/*z* 1119, a difference of 100 Da between *m*/*z* 1019 and *m*/*z* 1119 suggested 3-hydroxy-2-methylbutyric substituted on resin glycoside, ion at *m*/*z* 1001 [M − H − 118]^−^ and *m*/*z* 1019 [M − H − 3-hydroxy-2-methylbutyric residue]^−^ supported that speculation. Ions at *m*/*z* 937 [1019 − 82]^−^, 917 [937 − 102]^−^ coupled with *m*/*z* 835 were observed in MS/MS spectrum. According to fragment rules and information listed above, the tentative identification given to peak 37′, 49′, 52′, 56′ were isomer of *m*/*z* 853 + tiglic acid + 2-methylbutyric acid + 3-hydroxy-2-methylbutyric.

Peak 39′, 45′ exhibited [M + HCOO]^−^ at *m*/*z* 1139 in MS spectrum, [M − H]^−^ at *m*/*z* 1093 loss of C_2_H_4_O (44 Da) to form *m*/*z* 1049, product ions at *m*/*z* 1005 [M − H − 88]^−^, *m*/*z* 905 [M − H − 88 − 100]^−^, *m*/*z* 835 [M − H − 88 − 100 − 70]^−^ were checked in MS/MS spectrum, we inferred peak 39′, 45′ were isomer of *m*/*z* 853 + 3-hydroxy-2-methylbutyric + 2 mol isobutyric acid.

Peak 43′, 47′ showed [M − H]^−^ at *m*/*z* 1021, adduct ion [M + HCOO]^−^ at *m*/*z* 1067, ion at *m*/*z* 937 [M − H − (102 − H_2_O)]^−^ due to loss of 2-methylbutyric acid from deprotonated ion, *m*/*z* 919 [M − H − 102]^−^ with high abundance were obtained. Characteristic ion *m*/*z* 853 was attribute to successively loss of 2-methylbutyric acid residue from *m*/*z* 1021, *m*/*z* 561, *m*/*z* 417, *m*/*z* 399 combined with *m*/*z* 271 were observed. Therefore, peak 43′, 47′ were tentatively characterized as isomer of *m*/*z* 853 + 2-methylbutyric acid+2-methylbutyric acid.

Peak 44′, 50′ exhibited deprotonated ion at *m*/*z* 1117, [M + HCOO]^−^ ion at *m*/*z* 1163 was observed in MS spectrum. In MS/MS a predominant productive ion *m*/*z* 1017 [M − H − (118 − H_2_O)]^−^, coupled with *m*/*z* 999 [M − H − 118]^−^, *m*/*z* 917 [M − H − 118 − (100 − H_2_O)]^−^ and *m*/*z* 935 [M − H − 100 − 82]^−^. Ion at *m*/*z* 835 combined with *m*/*z* 561, *m*/*z* 417, *m*/*z* 399, *m*/*z* 271 from the cleavage of glycoside bond were checked. We thus inferred the structure of peak 44′ and 50′ was 2 mol tiglic acid + 3-hydroxy-2-methylbutyric substituted on parent drug *m*/*z* 853.

Peak 53′, 55′ were eluted at 91.147 min and 89.303 min, respectively. For structural elucidation, [M − H]^−^ at *m*/*z* 1119 and its adduct ion [M + HCOO]^−^ at *m*/*z* 1165, [M + Cl]^−^ at *m*/*z* 1155 were found in MS spectrum. In analysis of its MS/MS spectrum, ion at *m*/*z* 1019 [M − H − C_5_H_8_O_2_]^−^, *m*/*z* 1001 [M − H − 118]^−^ indicated the existence of 3-hydroxy-2-methylbutyric, *m*/*z* 919 [M − H − 118 − 82(100 − H_2_O)]^−^, *m*/*z* 835 [M − H − 118 − 82 − 84(102 − H_2_O)]^−^ with high abundance were generated. According to proposed fragment pattern of resin glycoside, we gave tentative characterization to *m*/*z* 1119 was *m*/*z* 853 + 3-hydroxy-2-methylbutyric + tiglic acid + 2-methylbutyric acid.

## 3. Materials and Methods

### 3.1. Chemicals and Reagents

Acetonitrile, methanol and formic acid (MS grade) were purchased from Fisher Scientific (Fair Lawn, NJ, USA). Deionized water was obtained from Watson’s (Hong Kong, China).

### 3.2. References

References Turpethic acids C and Turpethoside B were provided by Professor Xiaoyi Wei, Key Laboratory of Plant Resources Conservation and Sustainable Utilization, South China Botanical Garden, Chinese Academy of Sciences, Guangzhou, China.

### 3.3. Sample Preparation

The extract of *Convolvulus* *scammonia* L. was provided by Xinjiang Uygur Medicine Limited Liability Company (Urumqi, China). The sample (1.0 g) was dispersed in 10 mL 100% methanol, and extraction was performed on a water bath at 25 °C assisted with ultrasound for 40 min. The extract was centrifuged at 14,000 rpm for 7 min, leading to the supernatant used as the test solution.

### 3.4. HPLC Condition

Chromatographic separation was performed on Ultimate 3000 RSLC system (Dionex, Sunnyvale, CA, USA). An ACQ-UITY UPLC^®^ HSS T3 column (100 mm × 2.1 mm i.d., 1.8 mm, Waters, MA, USA) maintained at 40 °C was used. The mobile phase consisted of acetonitrile (A) and 0.1% formic acid–water (B) with a gradient elution of 5% B at 0–1 min, 5–9% B at 1–4 min, 9–18% B at 4–22 min, 18–88% B at 22–92 min, 88–95% B at 92–92.1 min and 95% B at 92.1–110 min. A flow rate of 0.15 mL/min was set and injection volume was 1.00 μL.

### 3.5. Mass Spectrometric Conditions

High-resolution MS data were recorded on a Q Exactive^TM^ PLUS hybrid Quadrupole-Orbitrap Mass Spectrometer equipped with a heated ESI source (Thermo Fisher Scientific, Waltham, MA, USA) operating in the negative mode. The HESI parameters were set as follows: spray voltage, −2.8 kV; Capillary temperature 320 °C; sheath gas pressure, 35 arb; aux gas pressure, 10 arb; spare gas pressure, 0 arb; probe heater temperature, 350 °C; s-lens RF, 55 V. The Orbitrap analyzer scanned over *m*/*z* 100–1500 at a resolution of 70,000 in full scan MS^1^, and dynamic mass ranges in MS^2^ with resolution set at 13,500. AGC target values for MS^1^ and MS^2^ were 5e^6^ and 1e^5^, respectively. Maximum injection time (IT) for MS^1^ was defined as 100 ms and 50 ms for MS^2^.

MS^2^ experiments were performed by high collision induced dissociation (HCD) to improve the sensitivity and offer more fragments information for structural elucidation at normalized collision energy (NCE) 10/30/50 V, with isolation window at 4.0 *m*/*z*. Dynamic exclusion allows each precursor ion to be selected 1 time before being excluded with 10 s duration. The HRMS full scan and HCD MS^2^ data were viewed and processed by Xcalibur 4.2. (Thermo Fisher Scientific, Waltham, MA, USA).

### 3.6. Mass Defect Filter Approach

Resin glycosides analogues usually shared with the similar core structure; characteristic compounds were generated via various substituents. Therefore, with increasing the number of these substituted groups, the decimal mass and integer mass of the molecular weight would show the linear or fixed relationship in exploring the interested precursor ions. In other words, the elemental compositions of these compounds would produce regular changes with the increase of their molecular weights. Thus, their molecular ions and the isotope distributions can be dynamically presented by preferred mathematical method [23,24,25,26].

The MDF strategy relies on the decimal mass shift to remove the interferences and to pick out the target ions for structural elucidation. Core substructure and different substituent combination were the two essential parts to realize the MDF approach. In the present study, Molecular Weight 872 Da (C_40_H_72_O_20_), MW 854 Da (C_40_H_70_O_19_), MW 870 Da (C_40_H_70_O_20_) were picked as parent drug filter, mass defect type was standard mass defect (Standard Mass Defect = exact mass − nominal mass). Elemental composition prediction of the detected components was based on the following settings: elements in use, C 0–70, H 0–110, O 0–30; mass tolerance, <5 ppm; adduct species [M + HCOO]^−^, [M − H]^−^. The High-resolution MS data were processed by MDF using Compound Discovery 3.2 (Thermo Scientific, Waltham, MA, USA).

## 4. Conclusions

In the present study, a sensitive and effective strategy for rapid identification of the characteristic structural analogues resin glycosides in *Convolvulus scammonia* extract has been developed by using MDF coupled to DPIs analysis on a hybrid Q-Orbitrap mass spectrometer. Compared with the chemical constituents reported on *Convolvulus scammonia* [10,11,12], this work enriched our knowledge about resin glycosides of *Convolvulus scammonia*, not only the number of consititues, but also the variety of structural. A total of 80 components were identified, among which 79 indicated hydroxy C_16_ fatty acid as main aglycone that was in accordance with phytochemistry study on *Convolvulus scammonia*. In addition, aglycone with hydroxy C_17_ fatty acid was discovered in *Convolvulus scammonia* for the first time. Furthermore, ion *m*/*z* 1151 and *m*/*z* 1169 had deoxyhexose–hexose–deoxyhexose–hexose oligoglycosides conjunction type were checked due to the minor components can be exposed for the signal-noise ratio improved by MDF approach. For DPIs analysis, the reference compounds turpethoside B in which the carboxyl group of (S)-12-hydroxy-pentadecanoic acid was linked to C-2 of Rha to form a macrolactone ring, but the carboxyl group of jalapinolic acid was linked to C-3 of Rha to form a macrolactone ring as common appeared in *Convolvulus scammonia*, therefore, the abundance of dignositic fragment ions was a little bit different. In a word, DPIs analysis can provide a criterion to classify the target constituents detected into certain chemical families.

Mass defect filtering-oriented identification of resin glycosides is definitely an effective strategy, there were more MDF methods, including multiple MDF, raster MDF, five-point screening MDF and polygonal MDF, developed for more accurate precursor ions filtered and different types of plant metabolites can be discriminated by sorts of MDF methods.

## Figures and Tables

**Table 1:** A summary of key NL and DFIs for structure elucidation.

References	Precursor Ion [M − H]^−^	Adduct Ions [M + Cl]^−^, [M + HCOO]^−^	Diagnostic Fragment Ions (*m*/*z*)	Neutral Loss (Da)
Turpethoside B	*m*/*z* 1265.65344	[M + Cl]^−^ *m/z* 1301.62927	*m*/*z* 853.42, *m*/*z* 835.43,	HCOOH: 46.01
			*m*/*z* 689.38, *m*/*z* 579.34,	H_2_O: 18.01
		[M + HCOO]^−^ *m/z* 1311.65771	*m*/*z* 561.69, *m*/*z* 543.32,	Tiglic acid: 100.05
			*m*/*z* 433.28, *m*/*z* 271.23.	2-Methylbutyric acid: 102.08
Tupethic acid C	*m*/*z* 1047.52417	–	*m*/*z* 593.35, *m*/*z* 574.45,	Rha: 146.06
			*m*/*z* 447.30, *m*/*z* 429.29,	Glc: 162.05, H_2_O: 18.01
			*m*/*z* 285.24, *m*/*z* 267.46.	

**Table 2:** Transformations on resin glycosides.

Substitutes	Mass Change, Da	Mass Defect Shift, mDa
+2-methylbutyric acid	+C_5_H_8_O, 84.0569	56.9
+tiglic acid	+C_5_H_6_O, 82.0413	41.3
+isobutyric acid	+C_4_H_6_O, 70.0413	41.3
+3-hydroxy-2-methylenebutyric	+C_4_H_4_O, 68.0257	25.7
+3-hydroxy-2-methylbutyric	+C_5_H_8_O_2_, 100.0519	51.9
+Methyl	+CH_3_, 14.01565	15.7

**Table 3:** Mass-range (Da)-mass defect(mDa) for known resin glycosides from *Convolvulus scammonia*.

Filters	Mass Change, Da		Mass Defect Shift, mDa
*m*/*z* 853, C_40_H_69_O_19_	+isobutyric acid	C_44_H_75_O_20_ (923)	0.485
	+tiglic acid	C_45_H_75_O_20_ (935)	0.485
	+2-methylbutyric acid	C_45_H_77_O_19_ (937)	0.500
	+isobutyric acid, tiglic acid	C_49_H_81_O_21_ (1005)	0.526
	+tiglic acid, tiglic acid	C_50_H_81_O_21_ (1017)	0.526
	+2-methylbutyric acid, tiglic acid.	C_50_H_83_O_21_ (1019)	0.542
*m/z* 869, C_40_H_69_O_20_	+2-methylbutyric acid, tiglic acid	C_50_H_83_O_22_ (1035)	0.537
*m/z* 871, C_40_H_71_O_20_	+CH3, 2-methylbutyric acid, tiglic acid	C_51_H_87_O_22_ (1051)	0.568

**Table 4:** Summary of the mass spectral data of characterized glycosidic acids in ESI negative ion mode.

No.	Rt min	Formula	ΔMass ppm	Parent Compounds *m*/*z*	Transformations	Composition Change	MS^n^
1	58.424	C_50_H_86_O_23_	1.62	871	3-hydroxy-2-methylbutyric	+C_10_H_14_O_3_	MS [M − H]^−^ 1053.55042
					Tiglic acid		MS^2^ 971, 953, 935, 871, 853, 835, 725, 707, 579, 561, 417, 399, 271
2	59.374	C_50_H_88_O_23_	0.66	871	2-methylbutyric acid	+C_10_H_16_O_3_	MS [M − H]^−^ 1055.56519
					3-hydroxy-2-methylbutyric acid		MS^2^ 971, 955, 953, 937,871, 853, 835, 725, 707, 579, 561, 417, 399, 271
3	59.504	C_55_H_94_O_26_	–0.41	887	3-hydroxy-2-methylbutyric	+C_15_H_22_O_6_	MS [M − H]^−^ 1169.59558
					3-hydroxy-2-methylbutyric		MS^2^ 969, 951, 887, 851,579, 561, 417, 399, 271
					Tiglic acid		
4	60.543	C_50_H_88_O_24_	0.84	871	3-hydroxy-2-methylbutyric acid	+C_10_H_16_O_4_	MS [M − H]^−^ 1071.55969
					3-hydroxy-2-methylbutyric acid		MS^2^ 953, 909, 871, 853, 835, 725, 707, 579, 561, 417, 399, 271
5	62.294	C_50_H_86_O_23_	0.69	871	3-hydroxy-2-methylbutyric	+C_10_H_14_O_3_	MS [M − H]^−^ 1053.54944
					Tiglic acid		MS^2^ 971,953, 935, 871, 853, 835, 725, 707, 579, 561, 417, 399, 271
6	63.494	C_50_H_88_O_23_	1.13	871	2-methylbutyric acid	+C_10_H_16_O_3_	MS [M − H]^−^ 1055.56555
					3-hydroxy-2-methylbutyric acid		MS^2^ 971, 955, 953, 937,871, 853, 835, 725, 707, 579, 561, 417, 399, 271
7	65.234	C_49_H_86_O_23_	1.75	871	isobutyric acid	+C_9_H_14_O_3_	MS [M − H]^−^ 1041.05454
					3-hydroxy-2-methylbutyric acid		MS^2^ 941, 923, 871, 853, 835, 725, 707,579, 561, 417, 399, 271
8	65.571	C_50_H_86_O_23_	1.04	871	3-hydroxy-2-methylbutyric	+C_10_H_14_O_3_	MS [M − H]^−^ 1053.54980
					Tiglic acid		MS^2^ 971, 953, 935, 871, 853, 835, 725, 707, 579, 561, 417, 399, 271
9	65.721	C_55_H_94_O_25_	0.58	871	Tiglic acid	+C_15_H_22_O_5_	MS [M − H]^−^ 1153.60181
					3-hydroxy-2-methylbutyric acid		MS^2^ 1053,953, 871, 853, 835, 725, 707, 579, 561, 417, 399, 271
					3-hydroxy-2-methylbutyric acid		
10	65.966	C_55_H_94_O_25_	0.68	871	Tiglic acid	+C_15_H_22_O_5_	MS [M − H]^−^ 1153.60205
					3-hydroxy-2-methylbutyric acid		MS^2^ 1053, 953, 871, 853, 835, 725, 707, 579, 561, 417, 399, 271
					3-hydroxy-2-methylbutyric acid		
11	67.468	C_55_H_94_O_25_	0.47	871	Tiglic acid	+C_15_H_22_O_5_	MS [M − H]^−^ 1153.60168
					3-hydroxy-2-methylbutyric acid		MS^2^ 1053, 953, 871, 853, 835, 725, 707, 579, 561, 417, 399, 271
					3-hydroxy-2-methylbutyric acid		
12	68.081	C_55_H_94_O_25_	1.10	871	Tiglic acid	+C_15_H_22_O_5_	MS [M − H]^−^ 1153.60242
					3-hydroxy-2-methylbutyric acid		MS^2^ 1053, 953, 871, 853, 835, 725, 707, 579, 561, 417, 399, 271
					3-hydroxy-2-methylbutyric acid		
13	70.587	C_55_H_94_O_25_	0.58	871	Tiglic acid	+C_15_H_22_O_5_	MS [M − H]^−^ 1153.60181
					3-hydroxy-2-methylbutyric acid		MS^2^ 1053, 953, 871, 853, 835, 725, 707, 579, 561, 417, 399, 271
					3-hydroxy-2-methylbutyric acid		
14	71.012	C_54_H_92_O_24_	1.30	871	Tiglic acid	+C_14_H_20_O_4_	MS [M − H]^−^ 1123.59126
					Isobutyric acid		MS^2^ 1023, 941, 871, 853, 835, 725, 707, 579, 561, 417, 399, 271
					3-hydroxy-2-methylbutyric acid		
15	71.260	C_49_H_84_O_22_	1.31	871	Tiglic acid	+C_9_H_12_O_2_	MS [M − H]^−^ 1023.53931
					Isobutyric acid		MS2 941, 871, 853, 725, 579, 561, 417, 399, 271
16	71.331	C_54_H_92_O_24_	0.98	871	Tiglic acid	+C_14_H_20_O_4_	MS [M − H]^−^ 1123.59167
					Isobutyric acid		MS^2^ 1023, 941, 871, 853, 835, 725, 707, 579, 561, 417, 399, 271
					3-hydroxy-2-methylbutyric acid		
17	74.490	C_55_H_94_O_24_	0.94	871	Tiglic acid	+C_15_H_22_O_4_	MS [M − H]^−^ 1137.60730
					2-methylbutyric acid		MS^2^ 1055, 955, 871, 853, 835, 725, 707, 579, 561, 417, 399, 271
					3-hydroxy-2-methylbutyric acid		
18	74.561	C_50_H_86_O_22_	0.04	871	2-methylbutyric acid	+C_10_H_14_O_2_	MS [M − H]^−^ 1037.55518
					Tiglic acid		MS^2^ 955, 937, 935, 871, 853, 835, 725, 707, 579, 561, 417, 399, 271
19	76.437	C_54_H_92_O_24_	0.20	871	Tiglic acid	+C_14_H_20_O_4_	MS [M − H]^−^ 1123.59082
					Isobutyric acid		MS^2^ 1023, 941, 871, 853, 835, 725, 707, 579, 561, 417, 399, 271
					3-hydroxy-2-methylbutyric acid		
20	76.632	C_50_H_88_O_22_	1.42	871	2-methylbutyric acid	+C_10_H_16_O_2_	MS [M − H]^−^ 1039.57056
					2-methylbutyric acid		MS^2^ 955, 871, 853, 835, 725, 707, 579, 561, 417, 399, 271
21	78.369	C_54_H_94_O_24_	0.95	871	isobutyric acid	+C_14_H_22_O_4_	MS [M − H]^−^ 1125.60742
					2-methylbutyric acid		MS^2^ 1025, 941, 871, 853, 835, 725, 707, 579, 561, 417, 399, 271
					3-hydroxy-2-methylbutyric acid		
22	79.754	C_55_H_94_O_24_	0.84	871	Tiglic acid	+C_15_H_22_O_4_	MS [M − H]^−^ 1137.60718
					2-methylbutyric acid		MS^2^ 1055, 955, 871, 853, 835, 725, 707, 579, 561, 417, 399, 271
					3-hydroxy-2-methylbutyric acid		

**Table 5:** Summary of the mass spectral data of characterized resin glycosides in ESI negative ion mode.

No.	Rt min	Formula	ΔMass ppm	Parent Compounds *m*/*z*	Transformations	Composition Change	MS^n^ *m*/*z*
1′	67.577	C_45_H_78_O_20_	1.51	853	2-methylbutyric acid	+C_5_H_8_O	MS [M + FA − H]^−^ 983.50909
							[M − H]^−^ 937.50262
							MS^2^ 853, 835, 579, 561, 417, 399, 271
2′	68.468	C_50_H_84_O_22_	1.17	853	Tiglic acid	+C_10_H_14_O_3_	MS [M + FA − H]^−^ 1081.54431
					3-hydroxy-2-methylbutyric acid		[M − H]^−^ 1035.54016
							MS^2^ 991, 953, 935, 853, 835, 717, 679, 661, 643, 579, 561, 417, 399, 271
3′	70.902	C_49_H_84_O_22_	1.24	853	Isobutyric acid	+C_9_H_14_O_3_	MS [M + FA − H]^−^ 1069.54492
					3-hydroxy-2-methylbutyric acid		[M − H]^−^ 1023.53955
							MS^2^ 935, 923, 835, 773, 671, 663, 579, 561, 417, 399, 271
4′	71.865	C_54_H_92_O_24_	1.52	853	Isobutyric acid	+C_14_H_22_O_5_	MS [M + FA − H]^−^ 1169.59766
					3-hydroxy-2-methylbutyric acid		[M − H]^−^ 1123.59155
					3-hydroxy-2-methylbutyric acid		MS^2^ 1079, 1035, 1023, 935, 835, 661, 635, 579, 561, 417, 399, 271
5′	72.129	C_50_H_84_O_22_	0.65	853	Tiglic acid	+C_10_H_14_O_3_	MS [M + FA − H]^−^ 1081.54419
					3-hydroxy-2-methylbutyric acid		[M − H]^−^ 1035.53955
							MS^2^ 991, 953, 935, 853, 835, 717, 679, 661, 643, 579, 561, 417, 399, 271
6′	73.185	C_50_H_84_O_22_	0.67	853	Tiglic acid	+C_10_H_14_O_3_	MS [M + FA − H]^−^ 1081.54431
					3-hydroxy-2-methylbutyric acid		[M − H]^−^ 1035.53943
							MS^2^ 991, 953, 935, 853, 835, 717, 679, 661, 643, 579, 561, 417, 399, 271
7′	73.657	C_50_H_86_O_22_	0.66	853	2-methylbutyric acid	+C_10_H_16_O_3_	MS [M + FA − H]^−^ 1083.55994
					3-hydroxy-2-methylbutyric acid		[M − H]^−^ 1037.55225
							MS^2^ 1019, 993, 937, 853, 835, 707, 663, 579, 561, 417, 399, 271
8′	73.905	C_50_H_84_O_22_	1.13	853	Tiglic acid	+C_10_H_14_O_3_	MS [M + FA − H]^−^ 1081.54480
					3-hydroxy-2-methylbutyric acid		[M − H]^−^ 1035.54114
							MS^2^ 991, 953, 935, 853, 835, 717, 679, 661, 643, 579, 561, 417, 399, 271
9′	74.610	C_50_H_86_O_23_	-0.31	853	3-hydroxy-2-methylbutyric acid	+C_10_H_16_O_4_	MS [M + FA − H]^−^ 1099.55291
					3-hydroxy-2-methylbutyric acid		[M − H]^−^ 1053.54980
							MS^2^ 1009, 909, 891, 853, 835, 737, 661, 579, 561, 417, 399, 271
10′	74.845	C_50_H_86_O_22_	-0.61	853	2-methylbutyric acid	+C_10_H_16_O_3_	MS [M + FA − H]^−^ 1083.55847
					3-hydroxy-2-methylbutyric acid		[M − H]^−^ 1037.55383
							MS^2^ 1019, 993, 935, 853, 835, 707, 663, 579, 561, 417, 399, 271
11′	74.977	C_50_H_84_O_22_	0.76	853	Tiglic acid	+C_10_H_14_O_3_	MS [M + FA − H]^−^ 1081.54431
					3-hydroxy-2-methylbutyric acid		[M − H]^−^ 1035.53748
							MS^2^ 991, 953, 935, 853, 835, 717, 679, 661, 579, 561, 417, 399, 271
12′	75.092	C_55_H_92_O_24_	0.65	853	Tiglic acid	+C_15_H_22_O_5_	MS [M + FA − H]^−^ 1181.59656
					3-hydroxy-2-methylbutyric acid		[M − H]^−^ 1135.59160
					3-hydroxy-2-methylbutyric acid		MS^2^ 1091, 1047, 1035, 991, 935, 853, 835, 661, 635, 579, 561, 417, 399, 271
13′	75.702	C_50_H_84_O_22_	0.50	853	Tiglic acid	+C_10_H_14_O_3_	MS [M + FA − H]^−^ 1081.54419
					3-hydroxy-2-methylbutyric acid		[M − H]^−^ 1035.53918
							MS^2^ 991, 953, 935, 853, 835, 717, 679, 661, 643, 579, 561, 417, 399, 271
14′	76.552	C_50_H_86_O_23_	0.87	853	3-hydroxy-2-methylbutyric acid	+C_10_H_16_O_4_	MS [M + FA − H]^−^ 1099.55505
					3-hydroxy-2-methylbutyric acid		[M − H]^−^ 1053.54810
							MS^2^ 1009, 909, 891, 853, 835, 737, 661, 579, 561, 417, 399, 271
15′	76.776	C_55_H_92_O_24_	1.46	853	Tiglic acid	+C_15_H_22_O_5_	MS [M + FA − H]^−^ 1181.59766
					3-hydroxy-2-methylbutyric acid		[M − H]^−^ 1135.59009
					3-hydroxy-2-methylbutyric acid		MS^2^ 1091, 1047, 1035, 991, 935, 853, 835, 661, 635, 579, 561, 417, 399, 271
16′	77.177	C_50_H_86_O_22_	0.42	853	2-methylbutyric acid	+C_10_H_16_O_3_	MS [M + FA − H]^−^ 1083.55969
					3-hydroxy-2-methylbutyric acid		[M − H]^−^ 1037.55469
							MS^2^ 1019, 993, 935, 853, 835, 707, 663, 579, 561, 417, 399, 271
17′	77.521	C_55_H_92_O_24_	1.08	853	Tiglic acid	+C_15_H_22_O_5_	MS [M + FA − H]^−^ 1181.59729
					3-hydroxy-2-methylbutyric acid		[M − H]^−^ 1135.59583
					3-hydroxy-2-methylbutyric acid		MS^2^ 1091, 1047, 1035, 991, 935, 853, 835, 661, 635, 579, 561, 417, 399, 271
18′	78.166	C_50_H_86_O_22_	1.12	853	2-methylbutyric acid	+C_10_H_16_O_3_	MS [M + FA − H]^−^ 1083.56042
					3-hydroxy-2-methylbutyric acid		[M − H]^−^ 1037.55444
							MS^2^ 1019, 993, 935, 853, 835, 707, 663, 579, 561, 417, 399, 271
19′	78.756	C_55_H_92_O_24_	0.92	853	Tiglic acid	+C_15_H_22_O_5_	MS [M + FA − H]^−^ 1181.59717
					3-hydroxy-2-methylbutyric acid		[M − H]^−^ 1135.58752
					3-hydroxy-2-methylbutyric acid		MS^2^ 1091, 1047, 1035, 991, 935, 853, 835, 661, 635, 579, 561, 417, 399, 271
20′	79.008	C_54_H_92_O_24_	1.27	853	Isobutyric acid	+C_14_H_22_O_5_	MS [M + FA − H]^−^ 1169.59766
					3-hydroxy-2-methylbutyric acid		[M − H]^−^ 1123.59155
					3-hydroxy-2-methylbutyric acid		MS^2^ 1079, 1035, 1023, 935, 835, 661, 635, 579, 561, 417, 399, 271
21′	79.452	C_55_H_92_O_24_	1.17	853	Tiglic acid	+C_15_H_22_O_5_	MS [M + FA − H]^−^ 1181.59729
					3-hydroxy-2-methylbutyric acid		[M − H]^−^ 1135.58594
					3-hydroxy-2-methylbutyric acid		MS^2^ 1091, 1047, 1035, 991, 935, 853, 835, 661, 635, 579, 561, 417, 399, 271
22′	79.881	C_50_H_84_O_22_	0.94	853	Tiglic acid	+C_10_H_14_O_3_	MS [M + FA − H]^−^ 1081.54468
					3-hydroxy-2-methylbutyric acid		[M − H]^−^ 1035.53992
							MS^2^ 991, 953, 935, 853, 835, 717, 679, 661, 643, 579, 561, 417, 399, 271
23′	79.903	C_55_H_92_O_24_	1.03	853	Tiglic acid	+C_15_H_22_O_5_	MS [M + FA − H]^−^ 1181.59729
					3-hydroxy-2-methylbutyric acid		[M − H]^−^ 1135.59265
					3-hydroxy-2-methylbutyric acid		MS^2^ 1091, 1047, 1035, 991, 935, 853, 835, 661, 635, 579, 561, 417, 399, 271
24′	80.623	C_55_H_94_O_24_	0.80	853	2-methylbutyric acid	+C_15_H_24_O_5_	MS [M + FA − H]^−^ 1183.61243
					3-hydroxy-2-methylbutyric acid		[M − H]^−^ 1137.60852
					3-hydroxy-2-methylbutyric acid		MS^2^ 1093, 1049, 1037, 991, 853, 661, 635, 579, 561, 417, 399, 271
25′	80.774	C_54_H_92_O_24_	1.35	853	Isobutyric acid	+C_14_H_22_O_5_	MS [M + FA − H]^−^ 1169.59766
					3-hydroxy-2-methylbutyric acid		[M − H]^−^ 1123.59155
					3-hydroxy-2-methylbutyric acid		MS^2^ 1079, 1035, 1023, 935, 835, 661, 635, 579, 561, 417, 399, 271
26′	81.148	C_55_H_92_O_24_	1.22	853	Tiglic acid	+C_15_H_22_O_5_	MS [M + FA − H]^−^ 1181.59705
					3-hydroxy-2-methylbutyric acid		[M − H]^−^ 1135.59314
					3-hydroxy-2-methylbutyric acid		MS^2^ 1091, 1047, 1035, 991, 935, 853, 835, 661, 635, 579, 561, 417, 399, 271
27′	81.305	C_54_H_90_O_23_	1.19	853	2-methylbutyric acid	+C_14_H_20_O_4_	MS [M + FA − H]^−^ 1151.58691
					3-hydroxy-2-methylbutyric acid		[M − H]^−^ 1105.58093
					3-hydroxy-2-methylenebutyric acid		MS^2^ 1061, 1005, 973, 961, 917, 835, 661, 635, 579, 561, 417, 399, 271
28′	81.463	C_55_H_92_O_24_	0.94	853	Tiglic acid	+C_15_H_22_O_5_	MS [M + FA − H]^−^ 1181.59741
					3-hydroxy-2-methylbutyric acid		[M − H]^−^ 1135.59253
					3-hydroxy-2-methylbutyric acid		MS^2^ 1091, 1047, 1035, 991, 935, 853, 835, 661, 635, 579, 561, 417, 399, 271
29′	82.021	C_55_H_94_O_24_	0.72	853	2-methylbutyric acid	+C_15_H_24_O_5_	MS [M + FA − H]^−^ 1183.61243
					3-hydroxy-2-methylbutyric acid		[M − H]^−^ 1137.60681
					3-hydroxy-2-methylbutyric acid		MS^2^ 1093, 1049, 1037, 991, 853, 661, 635, 579, 561, 417, 399, 271
30′	82.154	C_54_H_90_O_23_	1.17	853	2-methylbutyric acid	+C_14_H_20_O_4_	MS [M + FA − H]^−^ 1151.58679
					3-hydroxy-2-methylbutyric acid		[M − H]^−^ 1105.58411
					3-hydroxy-2-methylenebutyric acid		MS^2^ 1061, 1005, 961, 835, 661, 635, 579, 561, 417, 399, 271
31′	83.169	C_54_H_92_O_23_	0.92	853	Isobutyric acid	+C_14_H_22_O_4_	MS [M + FA − H]^−^ 1153.60205
					2-methylbutyric acid		[M − H]^−^ 1107.59619
					3-hydroxy-2-methylbutyric acid		MS^2^ 1063, 989, 905, 961, 835, 661, 635, 579, 561, 417, 399, 271
32′	83.256	C_55_H_94_O_24_	0.87	853	2-methylbutyric acid	+C_15_H_24_O_5_	MS [M + FA − H]^−^ 1183.61267
					3-hydroxy-2-methylbutyric acid		[M − H]^−^ 1137.60510
					3-hydroxy-2-methylbutyric acid		MS^2^ 1093, 1049, 1037, 991, 853, 661, 635, 579, 561, 417, 399, 271
33′	83.331	C_49_H_84_O_21_	1.90	853	2-methylbutyric acid	+C_9_H_12_O_2_	MS [M + FA − H]^−^ 1053.55078
					Isobutyric acid		[M − H]^−^ 1007.54419
							MS^2^ 923, 905, 919, 835, 773, 671, 663, 579, 561, 417, 399, 271
34′	83.788	C_55_H_94_O_24_	0.66	853	2-methylbutyric acid	+C_15_H_24_O_5_	MS [M + FA − H]^−^ 1183.61230
					3-hydroxy-2-methylbutyric acid		[M − H]^−^ 1137.60889
					3-hydroxy-2-methylbutyric acid		MS^2^ 1093, 1049, 1037, 991, 853, 661, 635, 579, 561, 417, 399, 271
35′	84.032	C_50_H_84_O_21_	1.74	853	2-methylbutyric acid	+C_10_H_14_O_2_	MS [M + FA − H]^−^ 1065.55054
					Tiglic acid		[M − H]^−^ 1019.54510
							MS^2^ 937, 919, 835, 661, 643, 579, 561, 417, 399, 271
36′	84.590	C_54_H_90_O_23_	1.20	853	Tiglic acid	+C_14_H_20_O_4_	MS [M + FA − H]^−^ 1151.58679
					isobutyric acid		[M − H]^−^ 1105.58105
					3-hydroxy-2-methylenebutyric acid		MS^2^ 1061, 1005, 961, 835, 661, 635, 579, 561, 417, 399, 271
37′	84.800	C_55_H_92_O_23_	1.01	853	Tiglic acid	+C_15_H_22_O_4_	MS [M + FA − H]^−^ 1165.60217
					2-methylbutyric acid		[M − H]^−^ 1119.59570
					3-hydroxy-2-methylbutyric acid		MS^2^ 1075, 1019, 1001, 937, 917, 835, 661, 635, 579, 561, 417, 399, 271
38′	84.863	C_49_H_84_O_21_	1.66	853	2-methylbutyric acid	+C_9_H_12_O_2_	MS [M + FA − H]^−^ 1053.55042
					Isobutyric acid		[M − H]^−^ 1007.54437
							MS^2^ 923, 905, 919, 835, 773, 671, 663, 579, 561, 417, 399, 271
39′	84.632	C_53_H_90_O_23_	0.63	853	Isobutyric acid	+C_13_H_20_O_4_	MS [M + FA − H]^−^ 1139.58594
					Isobutyric acid		[M − H]^−^ 1093.57764
					3-hydroxy-2-methylbutyric acid		MS^2^ 1049, 1005, 905, 835, 749, 661, 579, 561, 417, 399, 271
40′	85.717	C_50_H_84_O_21_	0.48	853	2-methylbutyric acid	+C_10_H_14_O_2_	MS [M + FA − H]^−^ 1065.54919
					Tiglic acid		[M − H]^−^ 1019.54413
							MS^2^ 937, 919, 835, 661, 643, 579, 561, 417, 399, 271
41′	85.850	C_55_H_92_O_24_	0.40	853	Tiglic acid	+C_15_H_22_O_5_	MS [M + FA − H]^−^ 1181.59656
					3-hydroxy-2-methylbutyric acid		[M − H]^−^ 1135.59082
					3-hydroxy-2-methylbutyric acid		MS^2^ 1091, 1047, 1035, 991, 935, 853, 835, 661, 635, 579, 561, 417, 399, 271
42′	86.207	C_54_H_90_O_23_	1.20	853	Tiglic acid	+C_14_H_20_O_4_	MS [M + FA − H]^−^ 1151.58618
					isobutyric acid		[M − H]^−^ 1105.58044
					3-hydroxy-2-methylenebutyric acid		MS^2^ 1061, 1005, 961, 835, 661, 635, 579, 561, 417, 399, 271
43′	86.989	C_50_H_86_O_21_	1.61	853	2-methylbutyric acid	+C_10_H_16_O_2_	MS [M + FA − H]^−^ 1067.56604
					2-methylbutyric acid		[M − H]^−^ 1021.55975
							MS^2^ 937, 919, 853, 835, 663, 579, 561, 417, 399, 271
44′	87.502	C_55_H_90_O_23_	0.41	853	Tiglic acid	+C_15_H_20_O_4_	MS [M + FA − H]^−^ 1163.58655
					Tiglic acid		[M − H]^−^ 1117.58057
					3-hydroxy-2-methylbutyric acid		MS^2^ 1099, 1073, 1017, 999, 937, 935, 917, 835, 661, 635, 579, 561, 417, 399, 271
45′	87.684	C_53_H_90_O_23_	0.55	853	Isobutyric acid	+C_13_H_20_O_4_	MS [M + FA − H]^−^ 1139.5863
					Isobutyric acid		[M − H]^−^ 1093.57959
					3-hydroxy-2-methylbutyric acid		MS^2^ 1049, 1005, 905, 835, 749, 661, 579, 561, 417, 399, 271
46′	87.994	C_54_H_90_O_23_	0.56	853	Tiglic acid	+C_14_H_20_O_4_	MS [M + FA − H]^−^ 1151.58618
					isobutyric acid		[M − H]^−^ 1105.58044
					3-hydroxy-2-methylenebutyric acid		MS^2^ 1061, 1005, 961, 835, 661, 635, 579, 561, 417, 399, 271
47′	88.589	C_50_H_86_O_21_	1.06	853	2-methylbutyric acid	+C_10_H_16_O_2_	MS [M + FA − H]^−^ 1067.56567
					2-methylbutyric acid		[M − H]^−^ 1021.55957
							MS^2^ 937, 919, 853, 835, 663, 579, 561, 417, 399, 271
48′	88.674	C_54_H_92_O_23_	0.58	853	Isobutyric acid	+C_14_H_22_O_4_	MS [M + FA − H]^−^ 1153.60168
					2-methylbutyric acid		[M − H]^−^ 1107.59656
					3-hydroxy-2-methylbutyric acid		MS^2^ 1063, 989, 905, 961, 835, 661, 635, 579, 561, 417, 399, 271
49′	89.303	C_55_H_92_O_23_	0.29	853	Tiglic acid	+C_15_H_22_O_4_	MS [M + FA − H]^−^ 1165.60132
					2-methylbutyric acid		[M − H]^−^ 1119.59595
					3-hydroxy-2-methylbutyric acid		MS^2^ 1075, 1037, 1001, 853, 835, 661, 635, 579, 561, 417, 399, 271
50′	89.328	C_55_H_90_O_23_	1.44	853	Tiglic acid	+C_15_H_20_O_4_	MS [M + FA − H]^−^ 1163.58691
					Tiglic acid		[M − H]^−^ 1117.58142
					3-hydroxy-2-methylbutyric acid		MS^2^ 1099, 1073, 1017, 937, 935, 891, 853, 835, 661, 635, 579, 561, 417, 399, 271
51′	90.649	C_54_H_92_O_23_	0.81	853	Isobutyric acid	+C_14_H_22_O_4_	MS [M + FA − H]^−^ 1153.60193
					2-methylbutyric acid		[M − H]^−^ 1107.59668
					3-hydroxy-2-methylbutyric acid		MS^2^ 1063, 1007, 1005, 961, 835, 661, 635, 579, 561, 417, 399, 271
52′	91.147	C_55_H_92_O_23_	0.91	853	Tiglic acid	+C_15_H_22_O_4_	MS [M + FA − H]^−^ 1165.60229
					2-methylbutyric acid		[M − H]^−^ 1119.59680
					3-hydroxy-2-methylbutyric acid		MS^2^ 1075, 1037, 1001, 853, 835, 661, 635, 579, 561, 417, 399, 271
53′	91.797	C_55_H_94_O_23_	0.59	853	2-methylbutyric acid	+C_15_H_24_O_4_	MS [M + FA − H]^−^ 1167.61743
					2-methylbutyric acid		[M − H]^−^ 1121.61243
					3-hydroxy-2-methylbutyric acid		MS^2^ 1077, 1021, 1019, 919, 853, 835, 661, 635, 579, 561, 417, 399, 271
54′	91.855	C_54_H_90_O_23_	1.39	853	2-methylbutyric acid	+C_14_H_20_O_4_	MS [M + FA − H]^−^ 1151.58716
					3-hydroxy-2-methylbutyric acid		[M − H]^−^ 1105.58069
					3-hydroxy-2-methylenebutyric acid		MS^2^ 1061, 1005, 961, 835, 661, 635, 579, 561, 417, 399, 271
55′	93.802	C_55_H_94_O_23_	0.66	853	2-methylbutyric acid	+C_15_H_24_O_4_	MS [M + FA − H]^−^ 1167.61743
					2-methylbutyric acid		[M − H]^−^ 1121.61145
					3-hydroxy-2-methylbutyric acid		MS^2^ 1077, 1021, 1019, 919, 853, 835, 661, 635, 579, 561, 417, 399, 271
56′	94.849	C_55_H_92_O_23_	1.09	853	Tiglic acid	+C_15_H_22_O_4_	MS [M + FA − H]^−^ 1165.60242
					2-methylbutyric acid		[M − H]^−^ 1119.59680
					3-hydroxy-2-methylbutyric acid		MS^2^ 1075, 1037, 1001, 853, 835, 661, 635, 579, 561, 417, 399, 271

## Data Availability

Not applicable.
